# Space-time clustering-based method to optimize shareability in real-time ride-sharing

**DOI:** 10.1371/journal.pone.0262499

**Published:** 2022-01-14

**Authors:** Negin Alisoltani, Mostafa Ameli, Mahdi Zargayouna, Ludovic Leclercq

**Affiliations:** 1 GeoTwin SAS, Paris, France; 2 Université Gustave Eiffel, COSYS-GRETTIA, Paris, France; 3 Université Gustave Eiffel, Université de Lyon, ENTPE, LICIT, Lyon, France; Central South University, CHINA

## Abstract

Real-time ride-sharing has become popular in recent years. However, the underlying optimization problem for this service is highly complex. One of the most critical challenges when solving the problem is solution quality and computation time, especially in large-scale problems where the number of received requests is huge. In this paper, we rely on an exact solving method to ensure the quality of the solution, while using AI-based techniques to limit the number of requests that we feed to the solver. More precisely, we propose a clustering method based on a new shareability function to put the most shareable trips inside separate clusters. Previous studies only consider Spatio-temporal dependencies to do clustering on the mobility service requests, which is not efficient in finding the shareable trips. Here, we define the shareability function to consider all the different sharing states for each pair of trips. Each cluster is then managed with a proposed heuristic framework in order to solve the matching problem inside each cluster. As the method favors sharing, we present the number of sharing constraints to allow the service to choose the number of shared trips. To validate our proposal, we employ the proposed method on the network of Lyon city in France, with half-million requests in the morning peak from 6 to 10 AM. The results demonstrate that the algorithm can provide high-quality solutions in a short time for large-scale problems. The proposed clustering method can also be used for different mobility service problems such as car-sharing, bike-sharing, etc.

## 1 Introduction

The growing pressure on urban transportation systems needs innovative solutions that can increase their efficiency. In recent years, new mobility services and intelligent transportation systems have shown the potential to be a part of the solution. Among these services, ride-sharing is becoming popular [1].

Ride-sharing originated as a general concept where individual travelers share a vehicle for a trip and split travel costs with others that have similar itineraries and time schedules [2]. Besides, transportation, like many other aspects of daily life, is being transformed by the information technology (IT) revolution [3]. The spread of mobile devices and the development of the Global Positioning System (GPS) make it possible for all the transport operators to adapt in real-time the transportation supply to travel demand [4]. These new technologies have made considerable changes in the transportation modes [5]. These options make it possible to have access to the vehicle’s position and perform the matching process of ride-sharing in real-time. These possibilities have led to the development and progress of a new type of ride-sharing, which is called real-time ride-sharing, also known as dynamic ride-sharing or *ad hoc* ride-sharing.

Dynamic ride-sharing refers to a system that supports an automatic ride-matching process between participants on very short notice or even en-route [6]. The application serves the passenger requests on the one hand, i.e., the mobility demand. On the other hand, it can impact the network capacity and can be affected by the network conditions. Thus, to assess the ride-sharing system, we need to assess both aspects. First, we have to find the best way to serve the network demand by matching the passengers and the fleet vehicles and solving a fleet management problem. Effective and efficient optimization technology that matches drivers and riders in real-time is one of the necessary components for a successful dynamic ride-share system [7]. Second, we have to assess the impact of the network condition on the ride-sharing system performance. Network congestion has impacts on the dynamic ride-sharing service. When the rides are executed, a gap can exist between the estimated travel times used by the optimization process and the travel times experienced. In assessing a dynamic ride-sharing system, it is essential to consider this gap.

Matching users to trips is very challenging in real-time since it must happen very quickly. In different researches on optimal assignment for ride-sharing, the problem is formulated as an integer linear programming problem, and then different approaches are taken to solve it [8]. The target is to reduce the computation time and obtain solutions close to the global optimum. [9] show that the computation time and the quality of the results are serious problems in the algorithms for optimizing shared mobility.

The underlying optimization problem in a ride-sharing service is a pickup and delivery problem with time windows (PDPTW) [10]. In [11, 12], the authors propose reviews of dynamic pickup and delivery problems, bringing up some interesting and unsolved questions, such as the optimal waiting strategies, the modifications of the objective function on a rolling basis, to name a few. It is shown that this problem is NP-hard [13]. Even simplified variants of the problem with a single-driver single-rider setting, single pickup, and drop off, or a single-objective function are still NP-hard [14]. Although there is vast literature on algorithms and solution methods for these problems, there is still room for improvement in these methods.

The number of requests for the mobility service at every time is huge in the large-scale problems. Therefore, the complexity of the solution method for the matching problem increases exponentially by a small increase in the service demand. It is substantial to consider that the patterns of demands and the patterns of supplies are spatially and temporally dependent [15]. Considering these dependencies, clustering methods can be applied to scale down the problems and make the computations faster.

Clustering methods enhance the scalability of matching methods by reducing the search space, making it possible to parallelize and balance the computing workload, and speeding up the matching computations.

In this paper, we propose a clustering method based on a “Shareability Function” (SF) that considers all the trips’ possible matching situations. Two trips can be shared either in parallel (passengers are in the same vehicle at some point) or in sequence [16].

In the previous studies, the clustering methods usually consider two close origins or two close destinations (closeness in terms of geographical position or announcement time) to share a trip, but in the proposed method, we consider all the possible sharing states for every two trips. So we can be sure that the matching system can explore all the sharing possibilities and the solution is close to the global optimum.

The shareability function computes the extra travel time that the vehicle has to spend to service each matching situation, compared to the situation where each trip is serviced independently without sharing. Then the shareability function for every two trips is the minimum computed value among the three different situations (parallel, sequence, and independence). So, when the shareability function is low for two specific requests, they have a high potential to be shared efficiently. We propose a clustering method to put the most shareable trips in separate clusters based on this new function. Then, we propose a heuristic method to solve the matching problem inside each cluster. The final algorithm can provide high-quality solutions in a short time for large-scale problems.

As discussed above, it is crucial to consider the interactions between the system and the network traffic in dynamic ride-sharing system evaluation. Also, it is important to consider other vehicles like personal cars besides the ride-sharing service vehicles in the network. In this paper, we define two different models to simulate the functioning of the proposed dynamic ride-sharing system: The “plant model”, based on Macroscopic Fundamental Diagram (MFD), is used to simulate the real traffic conditions and considers both service vehicles and personal vehicles in the network; The “prediction model”, based on the current mean speed, is used to calculate the travel times during the assignment process [16, 17].

To validate our approach, we employ the proposed method on the real data of Lyon city in France with half-million requests in the morning peak from 6 to 10 AM. To demonstrate the clustering method’s performance, we have compared the proposed method with two other clustering-based methods from the literature (spatial and temporal clustering). The results show that the method proposed in this paper can make a significant improvement in the quality of the solution and the computation time.

Since ride-shares are established on-demand, a ride-sharing system is similar to other on-demand forms of passenger transit such as taxis and dial-a-ride services like airport shuttles [2]. The clustering method proposed in this study can be used for other similar systems.

The contributions of this paper are listed below:

- We define three different states for sharing the trips, and a new shareability function is defined based on the extra travel time the vehicle has to pass to serve the shared trips.
- We present a new clustering method using both hierarchical and partitional clustering approaches to put the most shareable trips inside separate clusters.
- We apply an exact matching algorithm based on the branch-and-bound concept that considers both passengers’ and providers’ objectives and constraints.
- We define a new constraint to make the algorithm flexible for the decision-makers to choose different numbers of sharing.
- We define two different models to consider the network traffic impact on the system performance and the gap between the estimated travel times used by the optimization process and the travel times experienced.

The remainder of this paper is structured as follows. Section 2 is the literature review section on ride-sharing and clustering methods. Section 3 details our clustering method for dynamic ride-sharing. Section 4 defines our travelers matching algorithm. Section 5 describes the two simulation models used as plant and prediction models respectively. Section 6 provides the results of our experiments. Section 7 concludes this paper.

## 2 Literature review

In this section, we review the latest studies on different solution methods and clustering approaches for real-time and dynamic ride-sharing.

### 2.1 Solution methods for the ride-sharing problem

Due to the complexity of the dynamic ride-sharing problem, the exact solution methods are introduced to solve very small instances. The most frequently cited literature on PDPTW is [18], where they present a mixed linear integer programming formulation of PDPTW and a branch and cut solution for it. In another approach, the problem is formulated to a bi-objective linear programming model to minimize the number of vehicles and the total cost of the collaborative operation [19–21]. The authors in [22] later introduce an enhanced branch-and-cut-and-price solution to improve the solution further. These exact methods are usually used to solve static problems with deterministic data [18, 23, 24]. In the PDPTW, increasing the number of vehicles and passengers increases the dimension of solution space and computational time. The method proposed by [10] takes almost two hours to compute a case with 50 passengers and 15 vehicles (On an Intel Workstation running two Xeon E5-2680 processors clocked at 2.80 GHz with 20 cores and 192GB RAM running Windows Server 2008 x64 Edition).

There are different heuristic methods in the literature to solve the assignment problem [25, 26]. [27] propose an exact branch-and-cut algorithm for the Dial-a-Ride Problem (DARP) that can outperform the state-of-the-art solver CPLEX. The exact method is followed by a lean heuristic algorithm based on Large Neighborhood Search (LNS) for larger problem instances. In [28], the exact method is proposed to solve small instances of the problem. Then a Tabu search heuristic is proposed for the pick and delivery problems for ride-sharing.

Dynamic ride-sharing problem addresses short-term matching or even en-route matching [6]. This fact makes the assignment problem more complex. In some studies, researchers try to narrow the feasible solution space to make the computations faster and be able to assign the vehicles to the requests that are coming at each time to the system. For example, [29] present a method to tighten travelers’ time windows and eliminate unnecessary variables and constraints to narrow the solution space. [30] proposed a branch-and-bound algorithm for solving a real-time ride-sharing problem. They introduced a kinetic tree algorithm to schedule dynamic requests and adjust the routes on the fly. [29] proposed a branch-and-cut algorithm to solve a realistic DARP with multiple trips and request types and a heterogeneous fleet of vehicles. [31] proposed an online optimization method for the dial-a-ride problem in a multi-company setting.

Some researches have implemented meta-heuristic methods to solve the assignment problem [32–35]. [36] first used a genetic algorithm to solve the ride-matching problem and find a sub-optimal solution, and then they presented an insertion heuristic method to take care of the newly received requests by modifying the solution of the genetic algorithm when possible. [37] proposed hybrid-simulated annealing (HSA) method to assign passenger requests to shared taxis dynamically. [38] propose a hybrid Genetic Algorithm to solve the Heterogeneous Dial-a-Ride problem (HDARP). [6] introduced a rolling horizon approach that can provide high-quality solutions for dynamic ride-sharing systems where trip requests continuously enter the system.

Recently [9] has presented a survey of models and algorithms for optimizing shared mobility, and they have shown that one of the most critical problems in the solution for these systems is computation time and the quality of the solution. The critical point in solving a dynamic ride-sharing assignment problem is finding the potential trips that can be shared in real-time while the system is receiving the requests continually. The previous methods for ride-sharing matching usually devote the quality to save the computation time, as the matching process should occur very fast. The proposed methods usually miss a lot of sharing opportunities and get far from the optimal situation. Compared to the previous approaches, this paper proposes a method that combines the benefits of exact solving in terms of solution quality and the benefits of heuristics and meta-heuristics in terms of computation time and scalability. On the one side, we present an algorithm based on branch-and-bound that can provide the optimal solution for small instances of the problem. On the other side, we introduce a method based on clustering that groups the requests and put the most shareable trips in the same clusters. The exact algorithm is executed with the requests inside each cluster. A rolling horizon approach is introduced to handle the trip requests in real-time. We show that the clustering method provides high-quality solutions while reducing the computation time significantly. Furthermore, it is important to consider the impacts of the network on travel times and ride-sharing system performance. This point has been neglected in the previous studies. They usually consider static travel times in the assignment process [39]. A few studies have considered the impact of traffic conditions on ride-sharing [40]; however, there is no benchmark considering traffic conditions. In our method, we consider two different models for the dynamic ride-sharing system to consider the gap between the estimated and the realized travel times.

### 2.2 Clustering methods

In the literature, there are clustering methods to handle large-scale problems, like dividing the time into several time slots or dividing the space into several clusters, road segments, or cells [41, 42].

To address the ride-matching problem in large-scale configurations, [43] propose to partition the road network into distinct regions which represent certain sub-structures of the road network. [44] propose a clustering-based request matching and route planning algorithm whose basic operations are merging requested trips on road networks. [45] propose an algorithm that uses the dataset of taxi get-off points and performs a clustering of taxis on urban roads. They compare their method with classical clustering methods. However, the taxi clustering data in their study are conducted in a static environment. In [46], all pickup points are partitioned into several clusters, the vehicles dispatching and the ride-sharing problem are solved in each cluster. [47] show that an appropriate solution for large-scale problems is clustering the demand nodes and downsizing the network. To speed up the computation, Some researches try to limit the feasible region with clustering methods. They usually divide the demand nodes in the network into geographically dense clusters [48, 49]. In a recent study by [50], an extended insertion algorithm in conjunction with a Tabu search method is proposed, and a cluster-first-route-second approach is used to find heuristic solutions.

One of the recent researches on the clustering of the trips is done by [51]. They introduce the notion of a shareability network to quantify the spatial and temporal compatibility of individual trips in a dynamic environment. In their method, two trips are shareable if they would incur a delay of no more than five minutes. Then, [52] enrich the idea to model the sharing of vehicles instead of rides and address the minimum fleet problem in on-demanded urban mobility. In these clustering methods, the trips are clustered based solely on the situation of the origin points. However, in ride-sharing, other combinations of trips should be considered. In this paper, we propose the concepts of “sequential index” and “Shareability index” to assess the possibility of serving two trips with the same car in sequence or by sharing the trips. Our proposal employs a method that reduces the number of required vehicles.

In a recent study, [53] consider the combination of the ride-sharing and public transportation services and formulate a mixed integer programming model for the multimodal transportation planning problem. They propose a heuristic approach (i.e., angle-based clustering algorithm) and compare its efficiency with the exact solution for different settings. They show that the clustering algorithm works well both in small and large settings. However, the impact of the network traffic on the ride-sharing system performance is not considered. In this paper, we define two distinct models for the dynamic ride-sharing to calculate the travel times during the assignment and simulate real traffic conditions.

## 3 Clustering method for dynamic ride-sharing

### 3.1 Shareability function

To perform the clustering on the requests (*N*_*t*_) received by the system at time *t*, we define the “Shareability Function” (*SF*_*i*,*j*_) between request *i* and request *j* (∀*i*, *j* ∈ *N*_*t*_). This function computes the difference between the travel time when the two trips are shared and the travel time to serve each trip individually, for each pair of requests.

**Fig 1:**
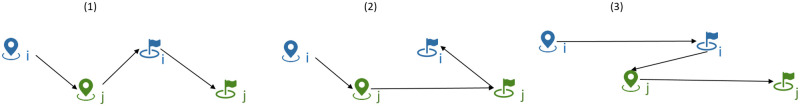
Trip situations.

In case of sharing, we consider three different situations for each pair of trips (Fig 1). In the first situation (1), two trips can be shared, and the vehicle first drops off the first passenger, and then it goes to the second passenger drop off (destination) point. In this situation, the travel time for the first passenger (*Tp*_*i*_) would be the summation of her/his waiting time (*Ws*_*i*_), the travel time between her/his origin and the next passenger origin (*TOO*_*i*,*j*_) and the travel time between the next passenger origin and her/his destination (*TOD*_*j*,*i*_). Similarly, the travel time for the second passenger (*Tp*_*j*_) would be the summation of her/his waiting time (*Ws*_*j*_), the travel time between her/his origin to the first passenger’s destination (*TOD*_*j*,*i*_) and the travel time from the first passenger’s destination and her/his destination (*TDD*_*i*,*j*_). So, *SF*_*i*,*j*_ (shareability function for trips *i* and *j*) when two trips have the first situation can be computed as in Eq 1.

$$\begin{array}{c} \begin{array}{cc} & Tp_{i}+Tp_{j}=Ws_{i}+TOO_{i,j}+Ws_{j}+TOD_{j,i}+TDD_{i,j}\\ & \forall i,j\in N\\ & SF_{i,j}^{1}=Tp_{i}+Tp_{j}-\left(TOD_{i,i}+TOD_{j,j}+W_{i}+W_{j}\right) \end{array} \end{array}$$ (1)

In situation (2), the second passenger is served by the service vehicle while the first passenger is on board. Thus, the travel time for the second passenger (*Tp*_*j*_) is the same as when served individually (summation of the waiting time and the time from the origin to the destination) and the travel time for the first passenger is the travel time of all the links from the first stop point (*O*_*i*_) to the last one (*D*_*j*_):

$$\begin{array}{c} \begin{array}{cc} & Tp_{i}+Tp_{j}=Ws_{i}+TOO_{i,j}+Ws_{j}+TOD_{j,j}+TDD_{j,i}\\ & \forall i,j\in N\\ & SF_{i,j}^{2}=Tp_{i}+Tp_{j}-\left(TOD_{i,i}+TOD_{j,j}+W_{i}+W_{j}\right) \end{array} \end{array}$$ (2)

In the third situation (3) that we consider for two trips, the trips are not shared, but the vehicle can serve two passengers sequentially [16]. This situation must be considered in the shareability index in order to encourage putting these trips in the same group while solving the optimization problem. The travel time for both passengers in this situation is the same as when they are served individually. But the vehicle travel time can decrease if the travel time between the first destination and the second origin is less than the summation of the travel time between the first origin and the closest depot and the travel time between the start depot and the second origin.

$$\begin{array}{c} \begin{array}{cc} & Tp_{i}+Tp_{j}=Ws_{i}+TOD_{i,i}+Ws_{j}+TOD_{j,j}\\ & \forall i,j\in N\\ & SF_{i,j}^{3}=Tp_{i}+Tp_{j}-\left(TOD_{i,i}+TOD_{j,j}+W_{i}+W_{j}\right)\\ & SF_{i,j}^{3}=Ws_{i}+Ws_{j}-\left(W_{i}+W_{j}\right) \end{array} \end{array}$$ (3)

The best situation for each two trips is the situation with minimum *SF*. So the algorithm chooses the condition that the additional travel time is minimum for sharing each pair of trips:

$$\begin{array}{c} \begin{array}{c} SF_{i,j}=\text{min}\left\{SF_{i,j}^{1},SF_{i,j}^{2},SF_{i,j}^{3}\right\} \end{array} \end{array}$$ (4)

### 3.2 Clustering based on a dissimilarity function

After computing the shareability function, we have the function value for each pair of requests that creates a “shareability matrix”. The shareability matrix is a dissimilarity matrix for the received requests: the higher the value, the least likely the two requests will be served together. In our approach, this matrix is used in the clustering process. We perform the clustering using the computed dissimilarity matrix. When we make clusters based on the *SF*, we put in the same cluster the trip requests that have more potential to be shared (the trips that have lower *SF*). There are two main categories of algorithms for clustering based on the shareability matrix, both are potentially relevant for our large-scale ride-sharing application:

1. Partitional clustering algorithms [54] cluster the data into *k* clusters. One of the usual algorithms for partitioned clustering is k-means clustering. K-means clustering is simple, fast, and flexible.
2. Hierarchical clustering methods in which the clusters are arranged in a tree-like structure. Hierarchical clustering can be divided into Agglomerative hierarchical clustering (AHC) and divisive clustering [55].

In [45], the authors have compared the hierarchical clustering and k-means clustering for urban taxi carpooling in a static environment. They show that compactness and separation are almost the same for hierarchical and k-means clustering for large cluster sizes. However, in dynamic large-scale problems, the results might be different. Besides, computation time becomes the critical criterion in this context. Thus, in our experiments, we implement both clustering methods and we assess their performance, considering both solutions quality and computation time.

#### 3.2.1 Multidimentional scaling and k-means clustering

K-Means method is a partitional clustering approach for decomposing the problem into independent subsets. It defines clusters of data based on their similarity, minimizing within-cluster variances. In the clustering procedure, the preferred number of clusters (*k*) should be specified in the algorithm before execution. The most common algorithm uses an iterative refinement technique. A common initialization step associates each observation (data point) with a cluster, and computes the set of cluster centroids as the mean of the observations of the cluster. Afterward, iterations will serve to optimize the clusters.

k-means clustering takes place based on the distance between points. Based on the study in [56], to be able to apply the appropriate k-mean clustering method, we need to convert the dissimilarity matrix into a distance matrix. Therefore, we use the multidimensional scaling method and change the shareability matrix into a distance matrix ([57]) (we have implemented the method using the mathtoolbox in C++).

After extracting the distance matrix, we are able to create the same size clusters for the data received at every assignment time step and put the most shareable trips in separate clusters using the modified k-means clustering method. This k-mean clustering is used to obtain clusters in preferred sizes [58]. Accordingly, considering the objective function value, we can find the best trade-off between cluster size and computation time. Putting the requests in clusters can also provide the opportunity for parallel computations. So the problem is divided into multiple sub-problems, and we favor a uniform distribution of requests among clusters to decrease computation times and facilitate parallel computations of each sub-problem.

#### 3.2.2 Hierarchical clustering

Hierarchical clustering offers a flexible and no-parametric approach and is an algorithm that builds hierarchy of clusters [59].

We use the agglomerative hierarchical method, which starts with taking singleton clusters (that contain only one request per cluster) at the bottom level and continue merging two clusters at a time to build a bottom-up hierarchy of the clusters [60].

## 4 Matching algorithm for dynamic ride-sharing

The matching algorithm aims to identify the travelers who can share their trips and assign them to a vehicle. In the assignment, it is important to consider both passengers’ and service provider’s objectives. According to the state-of-the-art, the most important operation objective for the service provider is to minimize the total travel time, and the total travel distance of vehicles [61, 62]. The passengers also need to get to the destination on time and have the minimum waiting time [63]. So we define the objective function for the matching algorithm as below where *i* is the index of passengers, and *m* is the index of vehicles, *W*_*i*_ is the waiting time for passenger *i*, *Tp*_*i*_ is the travel time for passenger *i*, *Tv*_*m*_ is the travel time for vehicle *m*, *D*_*m*_ is the travel distance for vehicle *m* and *α*, *β*, *γ*, and *δ* are the weights of each objective after normalization.

$$\begin{array}{c} \text{min}\sum_{i\in P}\left(\alpha .Ws_{i}+\beta .Tp_{i}\right)+\sum_{m\in M}\left(\gamma .Tv_{m}+\delta .D_{m}\right) \end{array}$$ (5)

The main constraints for the matching process are assignment constraints, time constraints, and capacity constraints. Assignment constraints are the very first constraints of the ride-sharing problem. Here, we make sure that the passenger is transported from the pick-up point to the drop-off point, and the pick-up point should be visited before the destination point. Also, we make sure that the same vehicle is handling a passenger pick-up and drop-off. We also have a flow constraint to ensure that the vehicle that enters a service node will also exit from it. Finally, we have considered a constraint to ensure that a vehicle is empty when it leaves the depot at the starting of the simulation. The assignment constraints are strict, and the algorithm should respect them all. In the time constraints, we define the earliest and latest pick-up and drop-off times for the passengers and ensure that the pick-up and drop-off times are inside these time windows. In the capacity constraints, we ensure that at each pick-up point, the demand does not exceed the vehicle’s capacity at that point. We also ensure that all the passengers who are picked up at the origin will be dropped off at the corresponding destination. In addition, we need to make sure that the passengers stay in the vehicle up to their destination. To make the algorithm flexible to choose different numbers of sharing, we have considered limitations on the number of sharing. The number of sharing for each passenger defines the allowed number of other passengers that can share their trip with this current passenger. So when the number of sharing is zero, it means that the service is not able to share, and we consider the in-sequence trips in the clustering function. But when we increase the number of sharing to one, it means that the service can serve two passengers simultaneously. Thus, the number of sharing constraint can make it possible to use the proposed algorithm for similar problems like ride-sourcing and dial-a-ride problems. In the end, we made sure that there was a sufficient number of vehicles in the fleet to serve all the requests.

**Fig 2:**
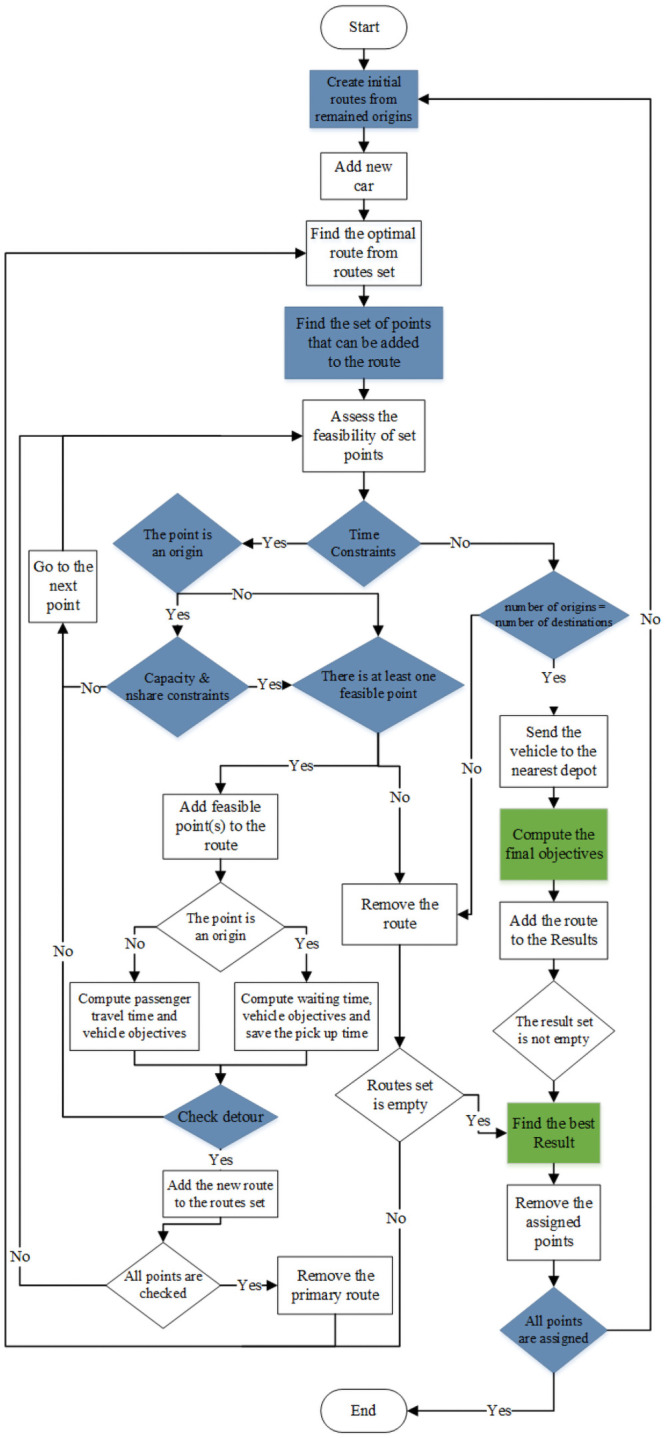
Final assignment algorithm.

The vehicle can be in two different situations. It is either circulating in the network to serve the on-board passengers (en-route vehicle) or waiting at a stop location for the new passengers (idle vehicle). Our proposed algorithm considers both situations. When the system receives a new request, it first checks the en-route vehicles that have not yet reached their maximal occupancy. So, the first part of the algorithm assigns the new requests in priority to en-route vehicles. The algorithm works to minimize the total travel time for both vehicles and passengers while respecting the capacity of the vehicle’s constraint and passengers’ time window constraint. The second part of the algorithm checks the idle vehicles waiting in depots to assign the passengers for the remaining requests. For this step, we introduce an exact method based on the branch-and-cut concept. The method creates branches of routes and then chooses the optimal solution among the feasible routes. Fig 2 shows this part of the algorithm. The blue boxes show the part of the algorithm that checks the constraints, and the green boxes show the part where we compute the objective function. The algorithm starts creating branches from the origin points and continues by adding the remaining origin points (in “create initial routes from remained origins”) and the related destinations. In “find the set of points that can be added to the routes”, we check the assignment constraints. So a destination point can be added to the route if and only if the related origin point has been already added to the route. Also, we check the capacity constraints, the time window constraints, and the number of sharing constraints. In the end, the algorithm computes the objective function for the feasible routes (if the number of origins and destinations are the same, and we are sure that all the destinations are visited) and chooses the route that has a minimum objective function. Then, we introduce a rolling horizon method to solve the problem dynamically. The requests that are assumed to be known are the only ones over the next rolling horizon (20 minutes). We also use an “assignment time horizon” of 10 minutes. That means that every 10 minutes, we execute our optimization algorithm while considering the requests of the next 20 minutes.

We present a small example with four requests to show the execution of the algorithm. Table 1 presents the requests. Each request has an associated number of requested seats, a maximum number of accepted sharing, an earliest pick-up time and a latest arrival time.

**Table 1:** Example with 4 requests (configuration).

Request	Travel distance	demand	nshare	EPT	LDT
1	19	1	3	8:00	8:45
2	11	2	2	8:00	8:25
3	24	2	1	8:15	9:00
4	18	2	3	8:30	9:20

**Fig 3:**
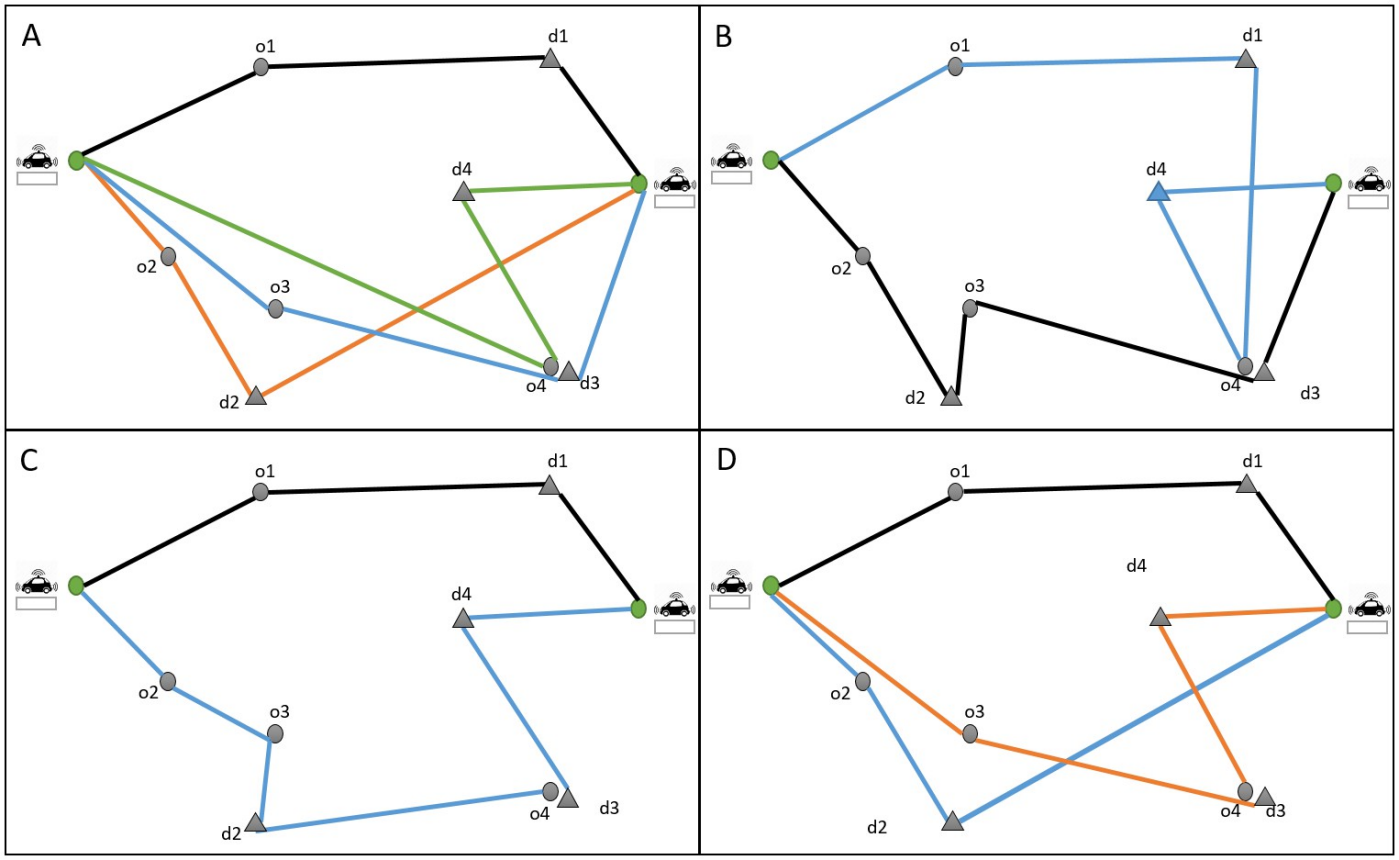
Feasible routes for the example.

The algorithm starts by creating branches of routes to serve the requests. It sends a car from the closest stop location to pick the passengers up at the origin point, and then it continues by adding the feasible points to the branches. First, the algorithm finds feasible branches. Fig 3 shows the final feasible solutions for the problem. The algorithm can find four feasible solutions for the problem.

Table 2 shows the total passengers’ waiting time, total vehicles’ travel distance, and the number of vehicles for these solutions (note that the waiting time for passengers is the difference between the passengers’ pick up time and the passengers’ desired departure time). Solution *A* is when a car serves each passenger separately without sharing. In this solution, the waiting time is minimum, and the passengers wait for 28 seconds to be picked up. Solution *B* serves passengers 1 and 4 with one car and passengers 2 and 3 with another car. It should be mentioned that in this solution, the trips are not shared. The first car serves passenger 1, and after dropping off this passenger, it goes to pick up passenger 4 at his destination. This solution increases the waiting time to 51 seconds, but it can reduce the travel distance from 3,030 meters to 2,085. Solution *C* serves the four requests with two cars like solution *B*. However, it shares the trip for passengers 2 and 3, and it can make more significant progress in reducing the travel distance to 1,785 meters. The waiting time with this solution is 53 seconds. Solution *D* increases the waiting time just 1 second compared to solution *A*. It serves requests 3 and 4 with a car in sequence (without sharing) and requests 1 and 2 with two cars separately. If the weights for all the objective functions are equal, after normalizing the objective functions, the optimal solution will be solution *C*.

**Table 2:** Example with 4 requests (solutions).

Solution	Total waiting time (s)	Total travel distance (m)	Number of cars
A	28	3030	4
B	51	2085	2
C	53	1785	2
D	29	2265	3

## 5 Simulation models for dynamic ride-sharing

The ride-sharing service’s optimization system uses estimates for the predicted travel time obtained from a so-called “prediction model”. When the fleet management plan is executed, a gap usually exists between the estimation and the real traffic condition. The so-called “plant model” requires dynamic adjustment of the initial assignment to fit with the conditions observed, and we use it to represent the real traffic condition.

To provide a realistic service, in the proposed simulation component of the dynamic ride-sharing system, we accurately distinguish the prediction and the plant models.

The trip-based MFD is used as the plant model to consider individual trips while keeping a very simple description of traffic dynamics. [17, 64, 65]. The general principle is to derive the inflow and outflow curves. When *n*(*t*) is the number of en-route vehicles at time *t* and the mean speed of travelers is *V*(*n*(*t*)) at every time *t*, the travel distance *L*_*i*_ by a car *i* entering at time *t* − *T*(*t*) must satisfy the following equation:

$$\begin{array}{c} L_{i}=\int_{t-T(t)}^{t}V\left(n\left(s\right)\right)ds \end{array}$$ (6)

The function *V*(*n*(*t*)) is the speed macroscopic fundamental diagram and can be derived from common observations for a transportation network [66]. For more details on the functioning of trip-based MFD, readers can refer to [67].

The prediction model estimates the traffic situation for the next assignment time horizon (every 10 minutes) to carry out travel time prediction during optimization. In our method, we assign the passengers to the cars based on this prediction. The prediction model is based on the direct travel time from each point *i* to *j* based on the *current mean speed* and the associated shortest path between the two points for the next 10 minutes. Then the optimization algorithm assigns all the requests for the next 10 minutes to the en-route cars or empty waiting cars.

In the rolling horizon method, the assignment procedure rolls over a specific horizon for the requests announced of a particular optimization step. As stated earlier, the rolling horizon in this paper is 20 minutes, and the optimization time step is 10 minutes. So, the requests of the next 20 minutes that have not yet been assigned are optimized every 10 minutes. Some requests are re-optimized every 10 minutes. If a trip has been assigned to a vehicle which has left the depot, the algorithm does not assign it again, but if a trip is in the schedule of a waiting vehicle, the algorithm places it in the set of optimized trips in the particular horizon and re-optimizes it.

In this method, every $\frac{TH}{2}$ time step, we stop the simulations and solve a new assignment problem for the requests over a new full rolling horizon (*TH*). Some requests may arrive just after the end of a simulation period, and this method prevents the system from being myopic to the new demand.

## 6 Experiments

### 6.1 Case study

**Fig 4:**
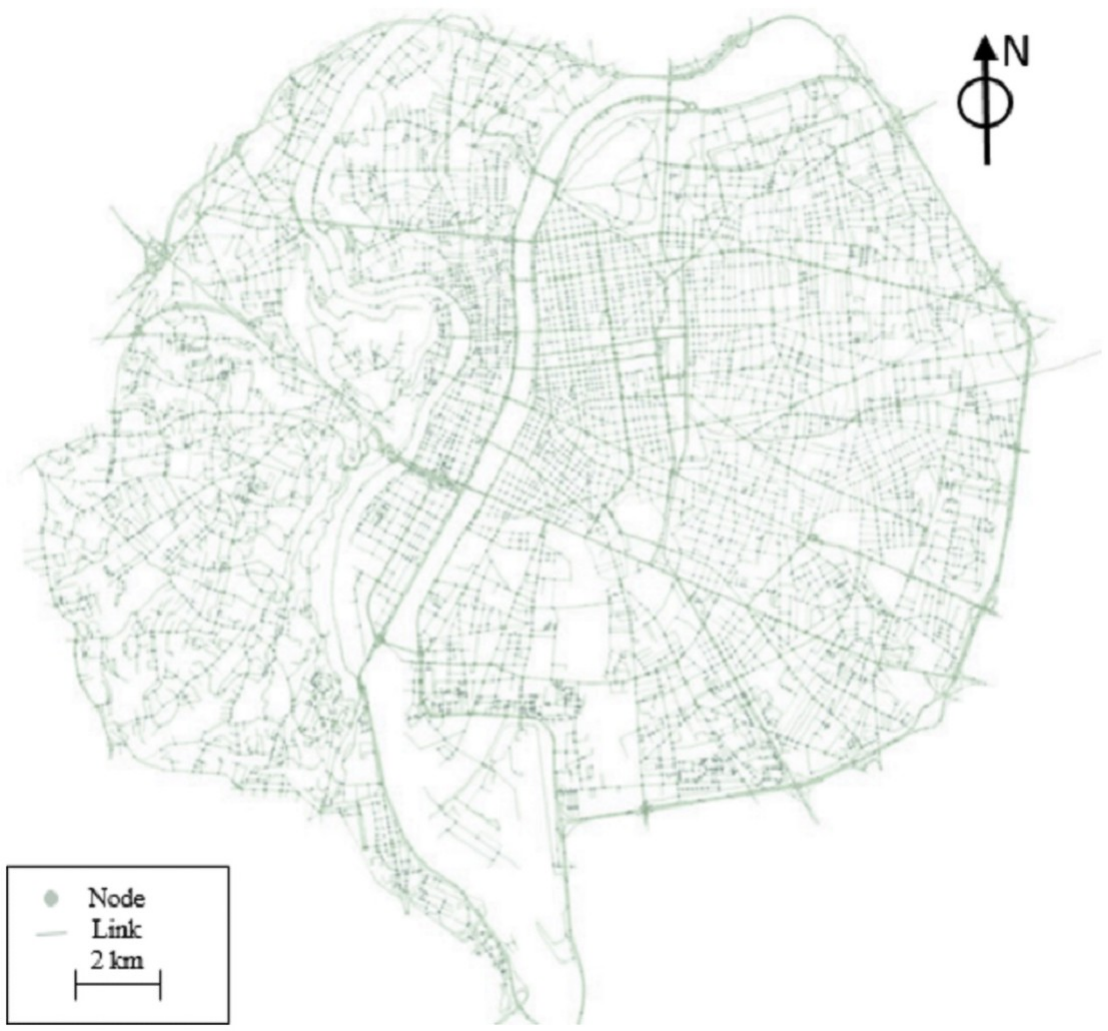
Network of Lyon.

In this paper, we use data from the network of Lyon city (the second-largest urban area of France) to evaluate the impact of proposed clustering method. The data set is available online at [68](https://research-data.ifsttar.fr/dataset.xhtml?persistentId=doi:10.25578/MLIDRM) The network area is more than 80 *km*^2^ and the origins/destinations (ODs) set contains 11,314 points. Fig 4 shows the network of the city.

The network is loaded with travelers of all ODs with a given departure time to represent the morning peak hour from 6 AM to 10 AM. The number of trips during this period is 484,690. We have 279,382 personal trips in the network and 205,308 demand for the service cars in the system.

The service provider has a fleet of vehicles in a ride-sharing system to serve the service requests. Participating service vehicles start up from a number of known locations or depots and after serving the assigned requests, they stop at this allowed locations to wait for the next passengers.

In our research, we define two kinds of depots: local depots and a central depot. The central depot in the network can feed all the local depots. On the one hand, distributing vehicles over the depots will decrease the waiting time for passengers. However, on the other hand, in the peak hour, if many vehicles are moving in the network, the congestion will increase, leading to more travel time for vehicles and passengers. We analyze the number of vehicles in depots over the network to decide about the best distribution for the vehicles.

To locate the cars at the beginning of the simulations, we use the historical data for the network demand to estimate the demand distribution over the network. Then we specify the number of cars at each location based on the demand for the depot. So if the demand is high, we consider more cars on the depot, and if the demand is low, we put fewer vehicles at that location.

**Fig 5:**
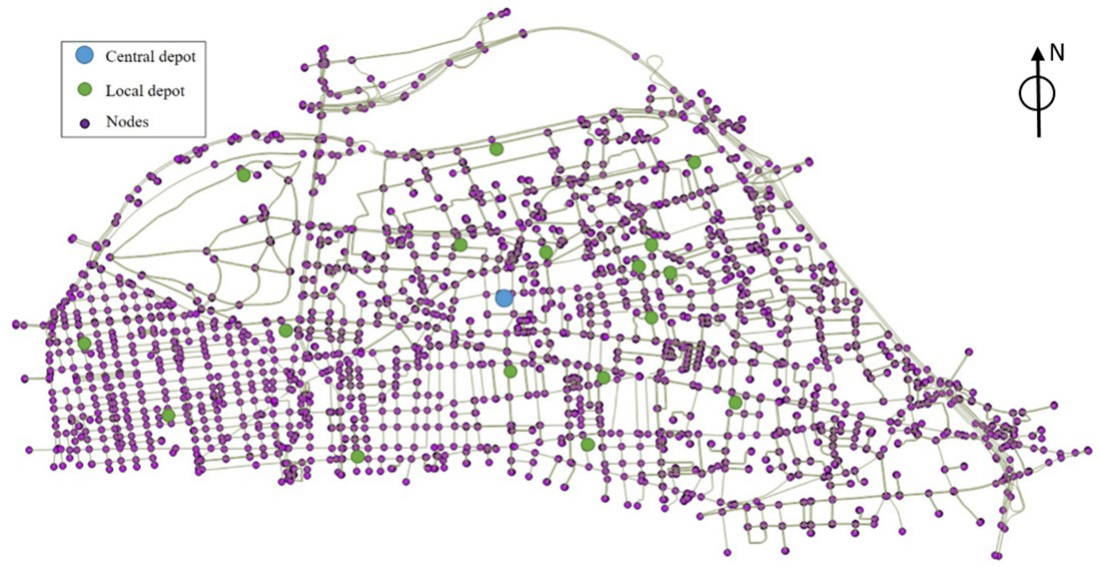
Central depot and local depots in a part of Lyon network.

In the network of Lyon city, we have defined nine central depots that are uniformly located in the network. The number of allowed stop locations is 2,272 points on the network. Fig 5 shows the location of one of the central depots and some local depots in the network of Lyon.

### 6.2 Sensitivity analyses on the optimization time step

**Fig 6:**
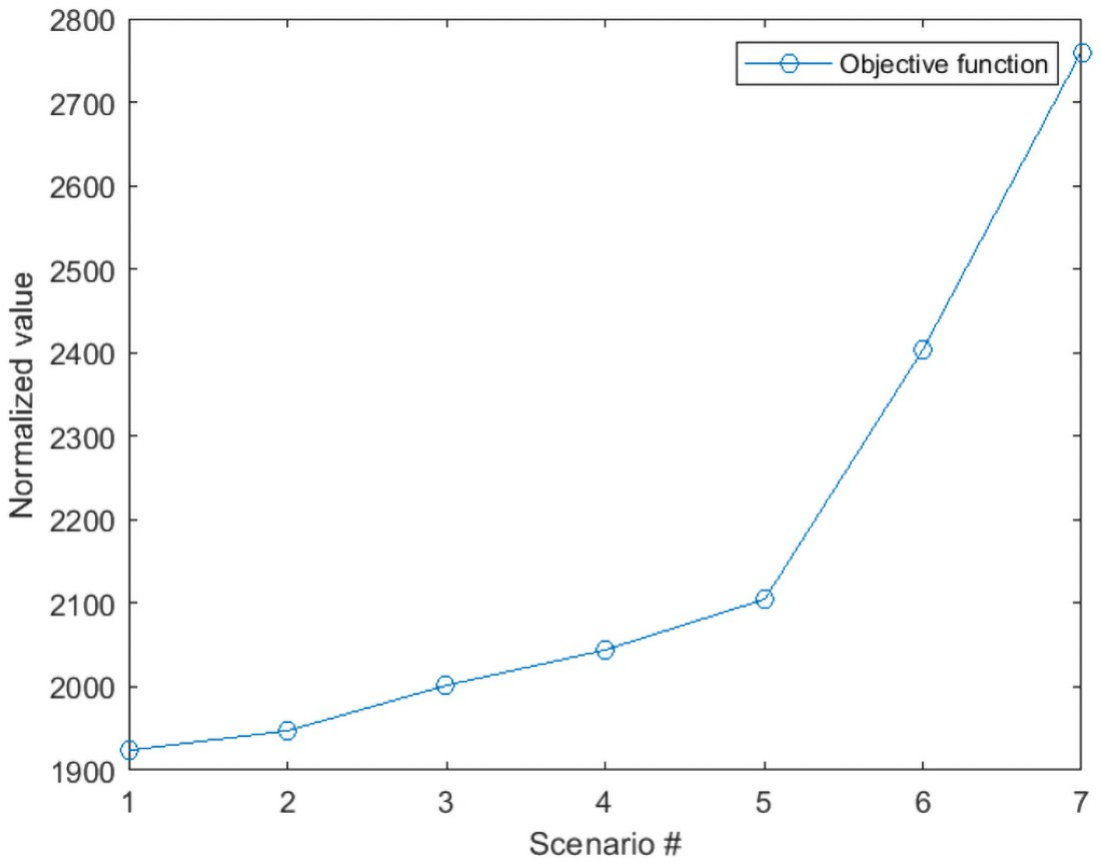
Objective function for different optimization times steps.

**Fig 7:**
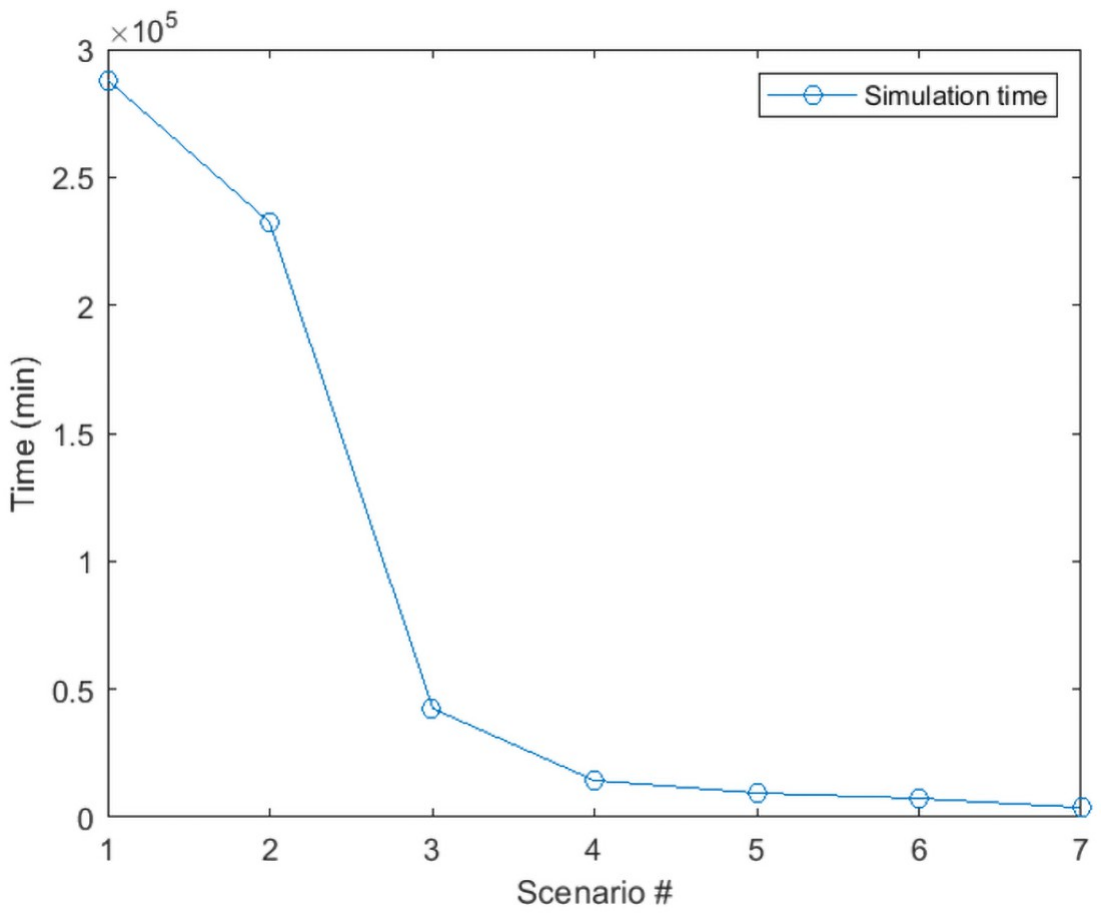
Simulation time for different optimization times steps.

As explained in section 4, considering all the requests over the full-time horizon can provide the global optimum solution for the ride-sharing problem. However, this greatly increases the number of variables and is not reasonable in practice. Also, in the dynamic ride-sharing system, the requests are announced in real-time, and the matching should take place en-route. To reduce the number of variables and bring the expression of the problem more in line with common practice, we implement a rolling horizon. The requests are assumed known only over the next rolling horizon. To choose the best trade-off between the simulation time and the objective function, we provide sensitivity analyses on different optimization and rolling horizon time steps. First, we optimized the problem without considering the rolling horizon approach using the exact solution method for 1092 requests. As the problem is NP-hard, the computation time is very high, even for small instances of the problem. However, we can have a reference to compare different optimization time steps having the optimal solution. Then we did the analyses with 120, 60, 40, 20, 10, and 4 minutes optimization time step (we choose the rolling horizon two times bigger than the optimization time step to avoid being myopic to the requests that may arrive exactly after the optimization time step). Table 3 presents the values for optimization time steps and rolling horizon. Figs 6 and 7 show the objective function and the simulation time for different rolling horizons (the first scenario is the optimal solution by solving the exact solution method over all the simulation horizon without considering rolling horizon approach).

**Table 3:** Optimization time steps and rolling horizon values.

Number	Optimization time step	Rolling horizon
1	-	-
2	120	60
3	60	30
4	40	20
5	20	10
6	10	5
7	4	2

The results show that decreasing the optimization time step can increase the objective function, but on the other hand, it reduces the simulation time significantly. The best trade-off between the computation time and the objective function is when the optimization time step is 10 minutes, and the rolling horizon is 20 minutes. We do the rest of the experiments using these values for the optimization time step and the rolling horizon.

### 6.3 Sensitivity analyses on the number of depots

The system sends a vehicle from the closest depot to the first origin. When the vehicle finishes all the assigned passengers’ trips, it goes back to the closest depot to wait for the next passengers. The location of local depots (allowed stop points for the service vehicles) can be selected from a set of feasible points on the network, such as taxi stations, some public parkings, and public transport stops. First, we select the locations of depots considering the Spatio-temporal distribution of demand. Then we analyze the demand for each depot to define the primary number of vehicles waiting on each depot at the beginning of the simulation. We define different scenarios to assess the impact of the number of local depots and the number of vehicles waiting in these locations. Considering these analyses, we choose the best number of depots and the distribution of vehicles over these depots at the beginning of the simulation horizon (early morning). The number of vehicles waiting at each point depends on the Spatio-temporal demand distribution around the stop location and the location capacity. It can vary from 0 to 5 vehicles. We have five different scenarios with 996, 1,450, 1,864, 2,272 and 2540 allowed stop locations. Table 4 shows the vehicles’ total travel time and distance and the passengers’ waiting time for different scenarios. The results show that increasing the number of depots can decrease the passengers’ waiting time and vehicles’ travel distance. But in the last scenario, as more vehicles are circulating in the network, the average speed decreases, so the vehicles’ total travel time increases.

**Table 4:** Simulation results for different number of depots.

Number of depots	Total passengers’ waiting time (h)	Total vehicles’ travel distance (km)	Total vehicles’ travel time (h)
996	1592.5	952447.0	30075.0
1450	1580.7	952305.0	30034.0
1864	1572.2	952220.0	30034.0
2272	1564.4	952139.0	30035.0
2540	1560.1	952039.0	30175.0

### 6.4 Determining the proper clustering method

As we said in section 3.2, we can use either the k-means clustering method or the hierarchical method to cluster the requests based on the presented shareability matrix. In our method, both the quality of the clustering method and the computation time are very important. The time complexity of k-means is linear, while that of hierarchical clustering is quadratic. Besides, k-means clustering requires prior knowledge of number *k* of clusters and also needs to use mutidimensional scaling to convert the similarity matrix into the distance matrix. On the other side, we can stop at whatever number of clusters we find appropriate in hierarchical clustering by interpreting the dendrogram. As we use the agglomerative hierarchical method, we can have larger clusters faster with the hierarchical method.

To choose the best clustering method, we have compared both methods considering the quality of objective function and the computation time for different sizes of problems.

Table 5 shows the objective function and computation time with k-means clustering and hierarchical clustering method for different sizes of problems.

**Table 5:** Clustering methods comparison.

Method	Number of requests	Objective function (normalized value)	Computation time (s)
Exact			
	112	29.99	12966.00
	1092	1924.00	288000.00
K-means clustering			
	112	30.96	100.10
	1092	1994.18	1134.00
	4482	7761.03	5340.00
	11160	20773.70	20981.10
Hierarchical clustering			
	112	30.96	99.03
	1092	1994.59	1131.10
	4482	7791.90	5187.50
	11160	20905.87	19950.00

To have a baseline for the comparisons, we have computed the optimal objective function (solution method without clustering) for 112 and 1,092 requests. In both clustering methods, we try to have clusters with 50 requests to ensure potential trips to be shared inside the clusters for both test cases with 50 requests and 1092 requests.

K-means clustering and hierarchical clustering methods increase the objective function by 3.22% for 112 requests and 3.64% and 3.66% for 1,092 requests, compared to the optimal solution. Both clustering methods can decrease the computation time from 216 minutes to less than 2 minutes. This shows that both clustering methods are very effective in terms of reducing the computation time while keeping the quality of the solution acceptable. Then, by increasing the number of requests, the computation time for both methods exponentially increases (the major part of the k-means method computation time is dedicated to the multidimensional scaling method, which exponentially increases by increasing the size of the problem).

K-means can give smaller objective function while hierarchical computation can result in a lower time. For 11,160 requests, the objective function is 0.64% lower for the k-means method, while the computation time is 5% more.

In our Lyon6 + Villeurbanne test case, the maximum number of requests is 11,235. So we can use k-means clustering for this test case to have better solutions. However, for the Lyon network, we have more than 200,000 service requests. So the hierarchical method is a better solution for this network, since it is faster as it works directly with the similarity matrix, and it can provide high-quality solutions.

### 6.5 The size of clusters

We try to have the same size clusters (to avoid too big or too small clusters) to keep the computation time low and have the opportunity for sharing in all the clusters.

There are different methods in the literature to choose the optimal size of clusters. Here, the quality of the clusters (how similar are points within a cluster) is very important. Furthermore, we have to be sure that the clusters are separated from each others, and the possibility of sharing two trips from two different clusters is minimum. Thus the best way to find the optimal size of clusters is to use the Sum of Squares method (SS) [69]. It is a clustering validation method that chooses the optimal size of clusters by minimizing the Within-cluster Sum of Squares (WSS) (a measure of how tight each cluster is) and maximizing the Between-cluster Sum of Squares (BSS) (a measure of how separated each cluster is from the others). We compute the WSS and BSS for all the clusters in different periods to evaluate the optimal size of clusters in different demand situations.

#### 6.5.1 K-means clustering

**Fig 8:**
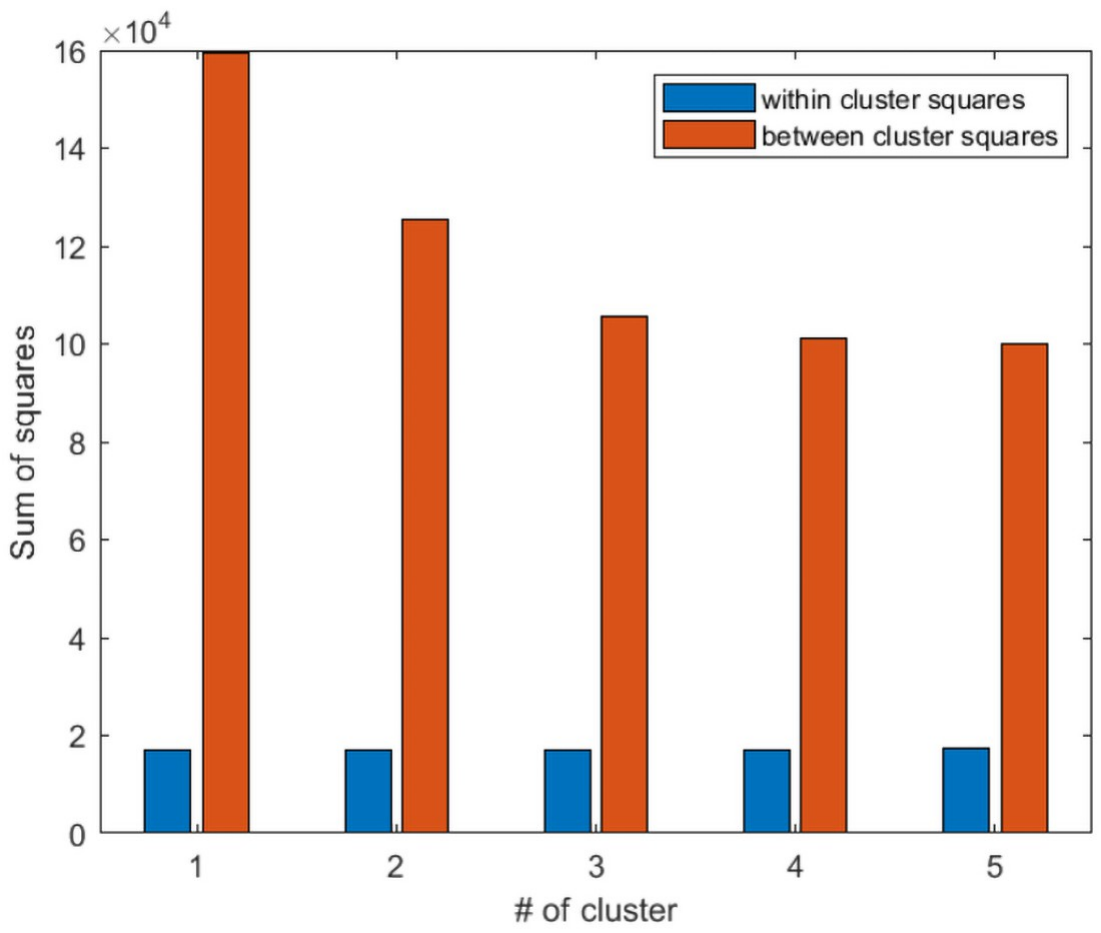
Sum of squares method for finding the optimal size of clusters in k-means clustering.

We compute the sum of squares for k-means clustering with cluster sizes from 10 to 50, presented in Table 6. Fig 8 shows that when the size of clusters is 30, we can find the best trade-off between WSS and BSS. So, we choose 30 as the size of clusters when we want to cluster the service requests with k-means clustering.

**Table 6:** Sum of Squares method for k-means clustering.

Number	Size of clusters
1	10
2	20
3	30
4	40
5	50

#### 6.5.2 Hierarchical clustering

In the hierarchical method, we can keep the first cluster of the desired size at the bottom of the dendrogram to have the same size clusters [70]. The crucial point here is to find an approximation for the size of clusters in the hierarchical method. As we have explained earlier, we use the hierarchical clustering when the number of demand is huge. So, we analyze the size of clusters from 75 to 300, presented in Table 7. Also, in large-scale networks with high levels of traffic congestion, the demand density is different in different times of a day (during the on-set and off-set of congestion). So we perform the analysis for the hierarchical clustering in four different times of the morning peak to be able to decide about the clusters size considering different conditions.

**Table 7:** Sum of squares method for hierarchical clustering.

Number	Size of clusters
1	75
2	100
3	125
4	150
5	175
6	200
7	225
8	250
9	275
10	300

**Fig 9:**
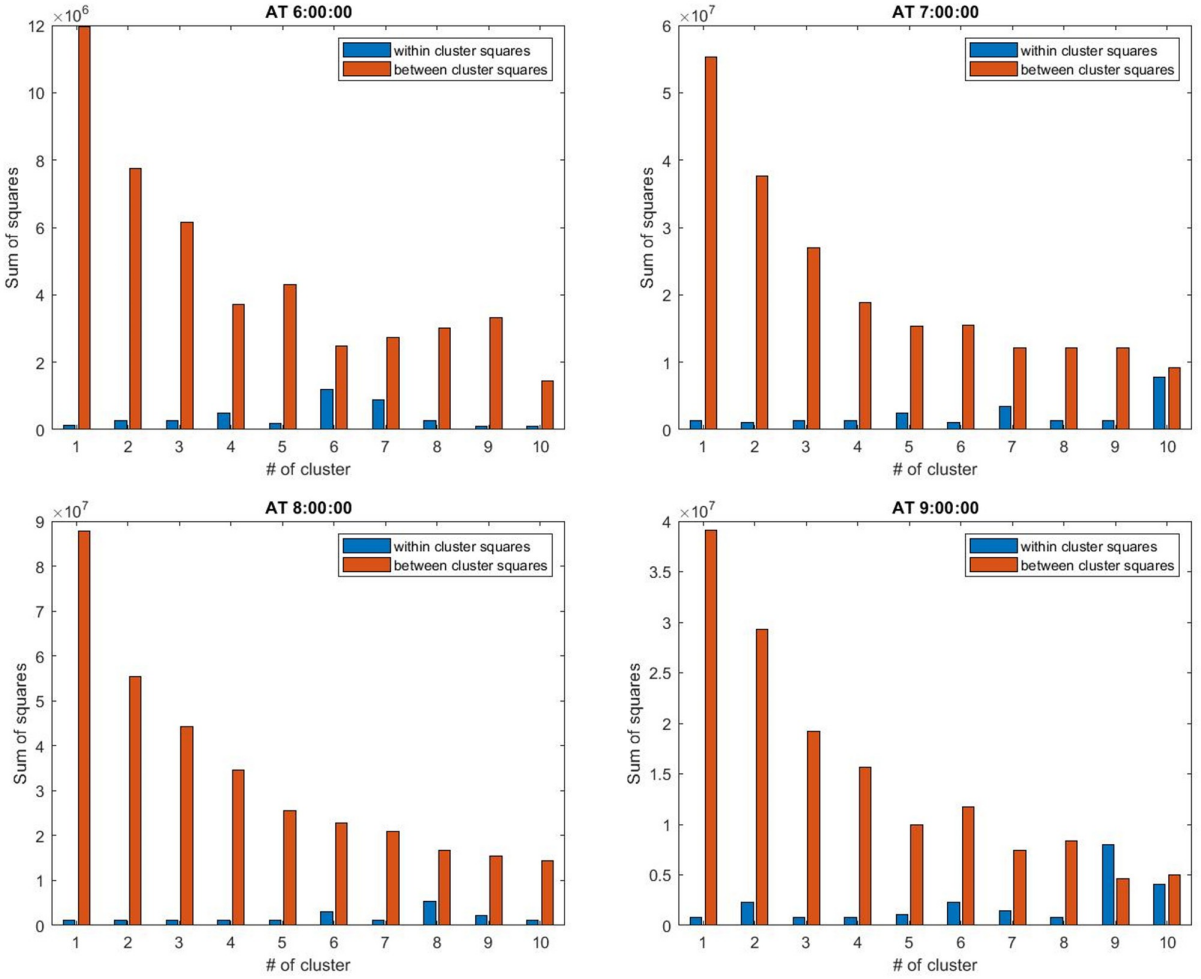
Sum of squares method for finding the optimal size of clusters in hierarchical clustering.

Fig 9 shows the SS method at different times of the simulations. Increasing the size of clusters decreases the BSS. It means that more number of clusters can ensure that the clusters are separate from each others. We have determined the cluster sizes that minimize the WSS and maximize the BSS. At 6 and 7 AM, the cluster sizes 100 and 125 can make this trade-off between WSS and BSS. At 8 and 9 AM, the best cluster sizes are 125 and 150. Therefore, the best cluster size to do the simulations for this scale is 125.

#### 6.5.3 Comparing the shareability clustering method with spatial and temporal methods

Some researches on ride-sharing use clustering methods to scale-down the matching problem and compute the assignment for the service vehicles faster. They usually divide the space geographically and use a spatial clustering to downsize the problem [46]. The important factor in spatial clustering is the distance between the trips’ origins. So two corresponding trips can be in the same cluster if the distance between their origins is small. Another approach is to cluster the trips based on the time in a temporal clustering method. In the temporal clustering, we put two trips is the same cluster based on their departure time and their position. Here, we compare the proposed clustering based on shareability function with spatial clustering and temporal clustering methods to show the quality of our proposal.

**Fig 10:**
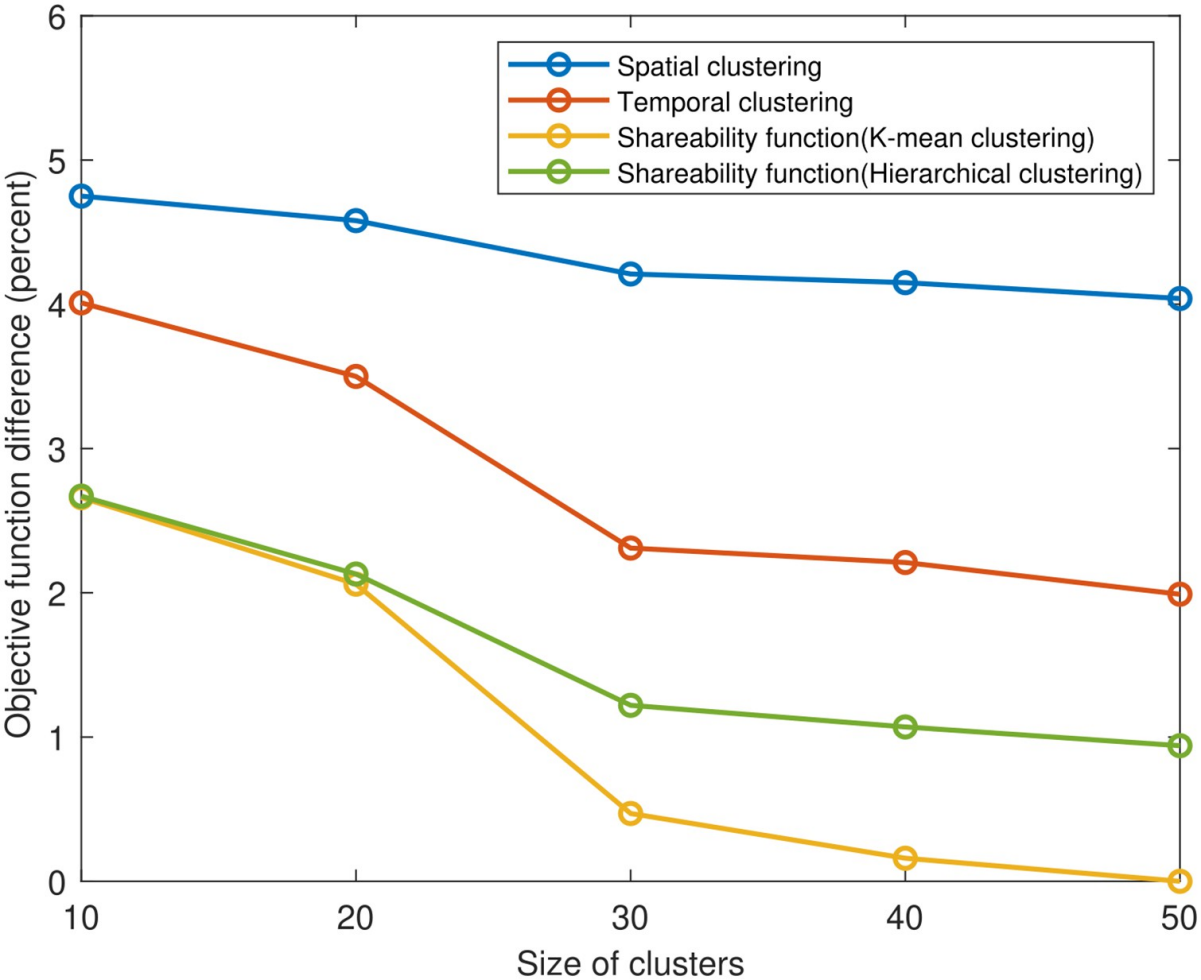
Comparing clustering methods’ objective function (market-share = 50%).

**Fig 11:**
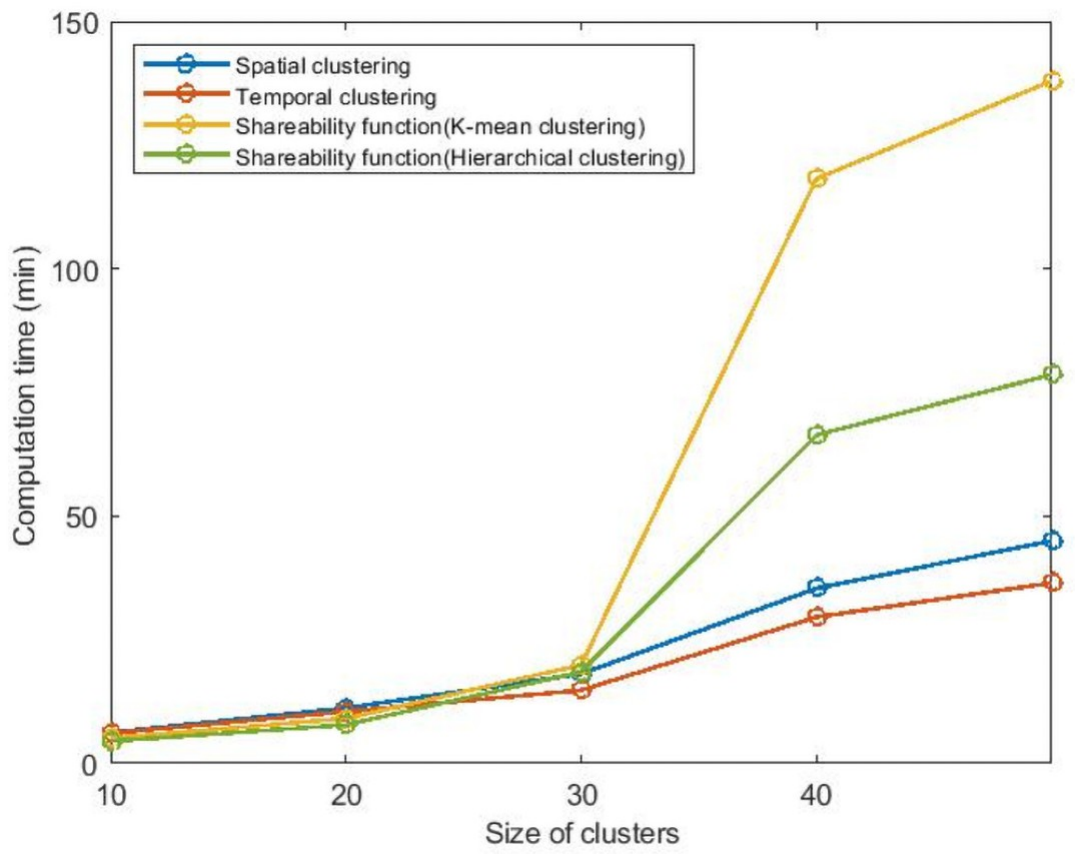
Comparing clustering methods’ computation time (market-share = 50%).

We compare the objective function and the computation time for the existing methods in the literature and our proposed shareability clustering method. Figs 10 and 11 show the comparison for five different cluster sizes when the market-share is 50% (market-share is the percentage of the total network demand that can be served by the ride-sharing service). Our proposed shareability function with k-means clustering method can provide the best objective function (when the size of clusters is 50 for this market-share). So the objective function for the shareability function and k-mean clustering is considered as a base, and the percentage of difference for other methods is computed considering this basic scenario in the first figure. The performance of spatial clustering is poor compared to the other methods. Even in the best situation, the spatial clustering’s objective function is 4.04% more than the shareability method with k-means clustering. The temporal clustering can perform better than spatial clustering, but it can not outperform our (space-time) shareability clustering method. The objective function for temporal clustering is 1.99% more than k-means clustering when the size of clusters is 50. As we have to convert the shareability matrix into a distance matrix using multidimensional scaling method to be able to use the k-means clustering, for big clusters (like cluster size of 50 here) the computation time increases exponentially. However, with the cluster size of 30, the algorithm can give a high-quality solution in a short time. As we can apply the hierarchical clustering directly on the shareability matrix to put the shareable trips in different clusters, the computation time for shareability function with hiearchical clustering is small. But the k-mean clustering can perform better in terms of quality of the clusters with our proposed shareability function. Choosing the proper clustering method for the shareability function highly depends on the scale of the problem and the demand density.

## 7 Conclusion

This paper presents a method to speed up the computations for the real-time ride-sharing assignment problem. The goal is to keep the solution’s quality and consider the impact of network traffic on the solution method performance. To this aim, we propose a new clustering method that, contrary to the existing clustering methods, considers all possible sharing situations. The clustering method is based on a space-time shareability function for large-scale real-time ride-sharing. We define three different states for sharing the trips, and the shareability function is computed based on the extra travel time the vehicle has to pass to serve the shared trips. The method puts the most shareable trips together inside the same clusters. We implemented both partitioned clustering (k-means clustering) and hierarchical clustering methods on the shareability matrix to cluster the requests. Then, we described an algorithm to solve the matching problem inside each cluster. The algorithm is based on the branch-and-bound concept that considers both passengers’ and providers’ objectives and constraints. In addition, we define two different models to consider the network traffic impact on the system performance and the gap between the estimated travel times used by the optimization process and the travel times experienced.

To evaluate the method, we employed the proposed method on the network of Lyon city in France with half-million requests in the morning peak from 6 to 10 AM. The results showed that the proposed clustering method produces high-quality solutions close to the optimal situation while significantly reducing computation time and can overcome the existing clustering methods.

Since ride-shares are established on-demand, a ride-sharing system is similar to other on-demand forms of passenger transit such as taxis and dial-a-ride services like airport shuttles. Thus, the clustering method proposed in this study can be used for other similar systems.

We have used earlier versions of this method to assess the impact of real-time ride-sharing on transportation networks [71]. In our future works, we plan to assess its impact on large-scale multi-modal networks, i.e., considering all available modes of transportation and not only private cars.

## References

[1] Tahmasseby S, Kattan L, Barbour B. Dynamic Real-Time Ridesharing: A Literature Review and Early Findings from a Market Demand Study of a Dynamic Transportation Trading Platform for the University of Calgary’s Main Campus; 2014.

[2] Furuhata M, Dessouky M, Ordóñez F, Brunet ME, Wang X, Koenig S. Ridesharing: The state-of-the-art and future directions. Transportation Research Part B: Methodological. 2013;57:28–46. doi: 10.1016/j.trb.2013.08.012

[3] Golob TF, Regan AC. Impacts of information technology on personal travel and commercial vehicle operations: research challenges and opportunities. Transportation Research Part C: Emerging Technologies. 2001;9(2):87–121. doi: 10.1016/S0968-090X(00)00042-5

[4] Zargayouna M, Othman A, Scemama G, Zeddini B. Multiagent simulation of real-time passenger information on transit networks. IEEE Intelligent Transportation Systems Magazine. 2018;12(2):50–63. doi: 10.1109/MITS.2018.2879166

[5] Altshuler T, Katoshevski R, Shiftan Y. Ride sharing and dynamic networks analysis. arXiv preprint arXiv:170600581. 2017;.

[6] Agatz NA, Erera AL, Savelsbergh MW, Wang X. Dynamic ride-sharing: A simulation study in metro Atlanta. Transportation Research Part B: Methodological. 2011;45(9):1450–1464. doi: 10.1016/j.trb.2011.05.017

[7] Agatz N, Erera A, Savelsbergh M, Wang X. Optimization for dynamic ride-sharing: A review. European Journal of Operational Research. 2012;223(2):295–303. doi: 10.1016/j.ejor.2012.05.028

[8] Zargayouna M, Zeddini B. Fleet organization models for online vehicle routing problems. In: Transactions on Computational Collective Intelligence VII. Springer; 2012. p. 82–102.

[9] Mourad A, Puchinger J, Chu C. A survey of models and algorithms for optimizing shared mobility. Transportation Research Part B: Methodological. 2019;. doi: 10.1016/j.trb.2019.02.003

[10] Mahmoudi M, Zhou X. Finding optimal solutions for vehicle routing problem with pickup and delivery services with time windows: A dynamic programming approach based on state–space–time network representations. Transportation Research Part B: Methodological. 2016;89:19–42. doi: 10.1016/j.trb.2016.03.009

[11] Berbeglia G, Cordeau JF, Laporte G. Dynamic pickup and delivery problems. European journal of operational research. 2010;202(1):8–15. doi: 10.1016/j.ejor.2009.04.024

[12] Pillac V, Gendreau M, Guéret C, Medaglia AL. A review of dynamic vehicle routing problems. European Journal of Operational Research. 2013;225(1):1–11. doi: 10.1016/j.ejor.2012.08.015

[13] Ropke S, Pisinger D. An adaptive large neighborhood search heuristic for the pickup and delivery problem with time windows. Transportation science. 2006;40(4):455–472. doi: 10.1287/trsc.1050.0135

[14] Gu QP, Liang JL, Zhang G. Algorithmic analysis for ridesharing of personal vehicles. Theoretical Computer Science. 2018;749:36–46. doi: 10.1016/j.tcs.2017.08.019

[15] Wang S, Li L, Ma W, Chen X. Trajectory analysis for on-demand services: A survey focusing on spatial-temporal demand and supply patterns. Transportation Research Part C: Emerging Technologies. 2019;108:74–99. doi: 10.1016/j.trc.2019.09.007

[16] Alisoltani N, Zargayouna M, Leclercq L. A Sequential Clustering Method for the Taxi-Dispatching Problem Considering Traffic Dynamics. IEEE Intelligent Transportation Systems Magazine. 2020;12(4):169–181. doi: 10.1109/MITS.2020.3014444

[17] Alisoltani N, Leclercq L, Zargayouna M, Krug J. Optimal fleet management for real-time ride-sharing service considering network congestion. In: TRB 2019, Transportation Research Board 98th Annual Meeting; 2019. p. 22p.

[18] Cordeau JF, Laporte G. The dial-a-ride problem: models and algorithms. Annals of operations research. 2007;153(1):29–46. doi: 10.1007/s10479-007-0170-8

[19] Wang Y, Zhang S, Guan X, Fan J, Wang H, Liu Y. Cooperation and profit allocation for two-echelon logistics pickup and delivery problems with state–space–time networks. Applied Soft Computing. 2021; p. 107528. doi: 10.1016/j.asoc.2021.107528

[20] Wang Y, Peng S, Guan X, Fan J, Wang Z, Liu Y, et al. Collaborative logistics pickup and delivery problem with eco-packages based on time–space network. Expert Systems with Applications. 2021;170:114561. doi: 10.1016/j.eswa.2021.114561

[21] Wang Y, Peng S, Zhou X, Mahmoudi M, Zhen L. Green logistics location-routing problem with eco-packages. Transportation Research Part E: Logistics and Transportation Review. 2020;143:102118. doi: 10.1016/j.tre.2020.102118

[22] Ropke S, Cordeau JF. Branch and cut and price for the pickup and delivery problem with time windows. Transportation Science. 2009;43(3):267–286. doi: 10.1287/trsc.1090.0272

[23] Baldacci R, Maniezzo V, Mingozzi A. An exact method for the car pooling problem based on lagrangean column generation. Operations Research. 2004;52(3):422–439. doi: 10.1287/opre.1030.0106

[24] Ghilas V, Cordeau JF, Demir E, Woensel TV. Branch-and-price for the pickup and delivery problem with time windows and scheduled lines. Transportation Science. 2018;52(5):1191–1210. doi: 10.1287/trsc.2017.0798

[25] Wang Y, Yuan Y, Guan X, Xu M, Wang L, Wang H, et al. Collaborative two-echelon multicenter vehicle routing optimization based on state–space–time network representation. Journal of Cleaner Production. 2020;258:120590. doi: 10.1016/j.jclepro.2020.120590

[26] Wang Y, Peng S, Xu M. Emergency logistics network design based on space–time resource configuration. Knowledge-Based Systems. 2021;223:107041. doi: 10.1016/j.knosys.2021.107041

[27] Braekers K, Kovacs AA. A multi-period dial-a-ride problem with driver consistency. Transportation Research Part B: Methodological. 2016;94:355–377. doi: 10.1016/j.trb.2016.09.010

[28] Wang X, Agatz N, Erera A. Stable matching for dynamic ride-sharing systems. Transportation Science. 2018;52(4):850–867. doi: 10.1287/trsc.2017.0768

[29] Liu M, Luo Z, Lim A. A branch-and-cut algorithm for a realistic dial-a-ride problem. Transportation Research Part B: Methodological. 2015;81:267–288. doi: 10.1016/j.trb.2015.05.009

[30] Huang Y, Jin R, Bastani F, Wang XS. Large scale real-time ridesharing with service guarantee on road networks. arXiv preprint arXiv:13026666. 2013;.

[31] Grootenboers F, De Weerdt M, Zargayouna M. Impact of competition on quality of service in demand responsive transit. In: German Conference on Multiagent System Technologies. Springer; 2010. p. 113–124.

[32] Li X, Hu S, Fan W, Deng K. Modeling an enhanced ridesharing system with meet points and time windows. PloS one. 2018;13(5):e0195927. doi: 10.1371/journal.pone.0195927 29715302 PMC5929516

[33] Aydin OF, Gokasar I, Kalan O. Matching algorithm for improving ride-sharing by incorporating route splits and social factors. PloS one. 2020;15(3):e0229674. doi: 10.1371/journal.pone.0229674 32130273 PMC7055897

[34] Qi X, Fu Z, Xiong J, Zha W. Multi-start heuristic approaches for one-to-one pickup and delivery problems with shortest-path transport along real-life paths. PloS one. 2020;15(2):e0227702. doi: 10.1371/journal.pone.0227702 32027655 PMC7004362

[35] Xia J, Curtin KM, Li W, Zhao Y. A new model for a carpool matching service. PloS one. 2015;10(6):e0129257. doi: 10.1371/journal.pone.0129257 26125552 PMC4488330

[36] Herbawi WM, Weber M. A genetic and insertion heuristic algorithm for solving the dynamic ridematching problem with time windows. In: Proceedings of the 14th annual conference on Genetic and evolutionary computation. ACM; 2012. p. 385–392.

[37] Jung J, Jayakrishnan R, Park JY. Dynamic shared-taxi dispatch algorithm with hybrid-simulated annealing. Computer-Aided Civil and Infrastructure Engineering. 2016;31(4):275–291. doi: 10.1111/mice.12157

[38] Masmoudi MA, Braekers K, Masmoudi M, Dammak A. A hybrid genetic algorithm for the heterogeneous dial-a-ride problem. Computers & operations research. 2017;81:1–13. doi: 10.1016/j.cor.2016.12.008

[39] Herbawi W, Weber M. The ridematching problem with time windows in dynamic ridesharing: A model and a genetic algorithm. In: Evolutionary Computation (CEC), 2012 IEEE Congress on. IEEE; 2012. p. 1–8.

[40] Wang X, Dessouky M, Ordonez F. A pickup and delivery problem for ridesharing considering congestion. Transportation letters. 2016;8(5):259–269.

[41] Davis N, Raina G, Jagannathan K. Taxi demand forecasting: A HEDGE-based tessellation strategy for improved accuracy. IEEE Transactions on Intelligent Transportation Systems. 2018;19(11):3686–3697. doi: 10.1109/TITS.2018.2860925

[42] Qi H, Liu P. Mining Taxi Pick-Up Hotspots Based on Spatial Clustering. In: 2018 IEEE SmartWorld, Ubiquitous Intelligence & Computing, Advanced & Trusted Computing, Scalable Computing & Communications, Cloud & Big Data Computing, Internet of People and Smart City Innovation (SmartWorld/SCALCOM/UIC/ATC/CBDCom/IOP/SCI). IEEE; 2018. p. 1711–1717.

[43] Pelzer D, Xiao J, Zehe D, Lees MH, Knoll AC, Aydt H. A partition-based match making algorithm for dynamic ridesharing. IEEE Transactions on Intelligent Transportation Systems. 2015;16(5):2587–2598. doi: 10.1109/TITS.2015.2413453

[44] Zuo H, Cao B, Zhao Y, Shen B, Zheng W, Huang Y. High-capacity ride-sharing via shortest path clustering on large road networks. The Journal of Supercomputing. 2020; p. 1–26.

[45] Qiang X, Shuang-Shuang Y. Clustering algorithm for urban taxi carpooling vehicle based on data field energy. Journal of Advanced Transportation. 2018;2018. doi: 10.1155/2018/3853012

[46] Chen S, Wang H, Meng Q. Solving the first-mile ridesharing problem using autonomous vehicles. Computer-Aided Civil and Infrastructure Engineering. 2020;35(1):45–60. doi: 10.1111/mice.12461

[47] Bard JF, Jarrah AI. Large-scale constrained clustering for rationalizing pickup and delivery operations. Transportation Research Part B: Methodological. 2009;43(5):542–561. doi: 10.1016/j.trb.2008.10.003

[48] Özdamar L, Demir O. A hierarchical clustering and routing procedure for large scale disaster relief logistics planning. Transportation Research Part E: Logistics and Transportation Review. 2012;48(3):591–602. doi: 10.1016/j.tre.2011.11.003

[49] Sáez D, Cortés CE, Núñez A. Hybrid adaptive predictive control for the multi-vehicle dynamic pick-up and delivery problem based on genetic algorithms and fuzzy clustering. Computers & Operations Research. 2008;35(11):3412–3438. doi: 10.1016/j.cor.2007.01.025

[50] Li Y, Chung SH. Ride-sharing under travel time uncertainty: Robust optimization and clustering approaches. Computers & Industrial Engineering. 2020;149:106601. doi: 10.1016/j.cie.2020.106601

[51] Santi P, Resta G, Szell M, Sobolevsky S, Strogatz SH, Ratti C. Quantifying the benefits of vehicle pooling with shareability networks. Proceedings of the National Academy of Sciences. 2014;111(37):13290–13294. doi: 10.1073/pnas.1403657111 25197046 PMC4169909

[52] Vazifeh MM, Santi P, Resta G, Strogatz S, Ratti C. Addressing the minimum fleet problem in on-demand urban mobility. Nature. 2018;557(7706):534. doi: 10.1038/s41586-018-0095-1 29795256

[53] Yu X, Miao H, Bayram A, Yu M, Chen X. Optimal routing of multimodal mobility systems with ride-sharing. International Transactions in Operational Research. 2021;28(3):1164–1189. doi: 10.1111/itor.12870

[54] Celebi ME. Partitional clustering algorithms. Springer; 2014.

[55] Vora P, Oza B, et al. A survey on k-mean clustering and particle swarm optimization. International Journal of Science and Modern Engineering. 2013;1(3):24–26.

[56] Paea S, Baird R. Information Architecture (IA): Using Multidimensional Scaling (MDS) and K-Means Clustering Algorithm for Analysis of Card Sorting Data. Journal of Usability Studies. 2018;13(3).

[57] Wang Q, Boyer KL. Feature learning by multidimensional scaling and its applications in object recognition. In: 2013 XXVI Conference on Graphics, Patterns and Images. IEEE; 2013. p. 8–15.

[58] Ganganath N, Cheng CT, Chi KT. Data clustering with cluster size constraints using a modified k-means algorithm. In: 2014 International Conference on Cyber-Enabled Distributed Computing and Knowledge Discovery. IEEE; 2014. p. 158–161.

[59] Murtagh F. A survey of recent advances in hierarchical clustering algorithms. The computer journal. 1983;26(4):354–359. doi: 10.1093/comjnl/26.4.354

[60] Reddy CK, Vinzamuri B. A Survey of Partitional and Hierarchical Clustering Algorithms. Data clustering: Algorithms and applications. 2013;87.

[61] Ota M, Vo H, Silva C, Freire J. STaRS: Simulating taxi ride sharing at scale. IEEE Transactions on Big Data. 2017;3(3):349–361. doi: 10.1109/TBDATA.2016.2627223

[62] Qian X, Zhang W, Ukkusuri SV, Yang C. Optimal assignment and incentive design in the taxi group ride problem. Transportation Research Part B: Methodological. 2017;103:208–226. doi: 10.1016/j.trb.2017.03.001

[63] Hyland MF, Mahmassani HS. Sharing Is Caring: Dynamic Autonomous Vehicle Fleet Operations Under Demand Surges; 2018.

[64] Mariotte G, Leclercq L, Laval JA. Macroscopic urban dynamics: Analytical and numerical comparisons of existing models. Transportation Research Part B: Methodological. 2017;101:245–267. doi: 10.1016/j.trb.2017.04.002

[65] Ameli M, Lebacque JP, Leclercq L. Cross-comparison of convergence algorithms to solve trip-based dynamic traffic assignment problems. Computer-Aided Civil and Infrastructure Engineering. 2020;35(3):219–240. doi: 10.1111/mice.12524

[66] Leclercq L, Chiabaut N, Trinquier B. Macroscopic fundamental diagrams: A cross-comparison of estimation methods. Transportation Research Part B: Methodological. 2014;62:1–12. doi: 10.1016/j.trb.2014.01.007

[67] Mariotte G, Leclercq L. Flow exchanges in multi-reservoir systems with spillbacks. Transportation Research Part B: Methodological. 2019;122:327–349. doi: 10.1016/j.trb.2019.02.014

[68] Ameli M, Alisoltani N, Leclercq L. Lyon Metropolis realistic trip data set including home to work trips with private vehicles during the morning peak; 2021. Available from: 10.25578/MLIDRM.

[69] Krzanowski WJ, Lai Y. A criterion for determining the number of groups in a data set using sum-of-squares clustering. Biometrics. 1988; p. 23–34. doi: 10.2307/2531893

[70] Hippocamplus. *Clustering into same size clusters*; 2018. Available from: http://jmonlong.github.io/Hippocamplus/2018/06/09/cluster-same-size/.

[71] Alisoltani N, Leclercq L, Zargayouna M. Can dynamic ride-sharing reduce traffic congestion? Transportation research part B: methodological. 2021;145:212–246. doi: 10.1016/j.trb.2021.01.004

